# Advancing rare disease therapeutics through digital twins: Opportunities in drug development and precision dosing

**DOI:** 10.1016/j.csbj.2025.11.047

**Published:** 2025-11-23

**Authors:** Charlotte Maria Ursula Dette, Veronika Alberg, Simeon Rüdesheim, Dominik Selzer, Fatima Zahra Marok, Nicola Luigi Bragazzi, Laura Maria Fuhr, Søren Brunak, Ewan R. Pearson, Tobias Zahn, Dimitra Kiritsi, Matthias Schwab, Thorsten Lehr

**Affiliations:** a Clinical Pharmacy, Saarland University, Saarbrücken 66123, Germany; b Dr. Margarete Fischer-Bosch-Institute of Clinical Pharmacology, Stuttgart 70376, Germany; c Novo Nordisk Foundation Center for Protein Research, Faculty of Health and Medical Sciences, University of Copenhagen, Copenhagen, Denmark; d Division of Diabetes, Endocrinology and Reproductive Biology, Ninewells Hospital and School of Medicine, University of Dundee, Dundee, UK; e Crowd Pharma GmbH, Pforzheim 75179, Germany; f Department of Dermatology, Medical Center – University of Freiburg, Faculty of Medicine, University of Freiburg, Freiburg 79106, Germany; g First Department of Dermatology, Faculty of Medicine, Aristotle University of Thessaloniki, Thessaloniki, Greece; h Departments of Clinical Pharmacology, Pharmacy and Biochemistry, University of Tübingen, Tübingen 72076, Germany; i Cluster of Excellence iFIT (EXC2180) “Image-Guided and Functionally Instructed Tumor Therapies”, University of Tübingen, Tübingen 72076, Germany

**Keywords:** Digital twins, Rare diseases, Orphan diseases, Orphan drug development, Recessive dystrophic epidermolysis bullosa

## Abstract

Rare disease(s) (RD/RDs) are typically characterized by (i) genetically driven chronic, and life-threatening disease progression, (ii) delayed diagnoses, (iii) limited treatment options, and (iv) substantial economic burdens due to direct and indirect medical costs. Challenges in RD research include limited patient populations, sparse disease data, poorly understood pathophysiology and reduced trial funding for new exploratory therapies. In recent years, digital twin(s) (DT/DTs) are increasingly used for patient care, disease management, and resource optimization. They serve as virtual replicas of individual patients that enable simulation, prediction, and optimization of outcomes through real-time data integration and can facilitate advancements in treatment outcome and prediction of disease progression leveraging model-based personalized predictions. This review included 16 studies and focuses on how DTs are currently used in RD research by analyzing the underlying modeling techniques, including physiologically based pharmacokinetic (PBPK) modeling, population pharmacokinetic (PopPK) modeling, quantitative systems pharmacology (QSP) modeling, physiome modeling, and combined approaches. It identifies the limitations of these models that currently prevent them from qualifying as true DTs. Furthermore, this review discusses the potential advantages of DTs in drug development for new treatment strategies, disease progression modeling, and clinical decision support for RD research. Finally, it outlines the current state of DT implementation in the RD field, revealing that DT implementation remains in an early stage of development.

## Graphical Abstract

**Figure:**
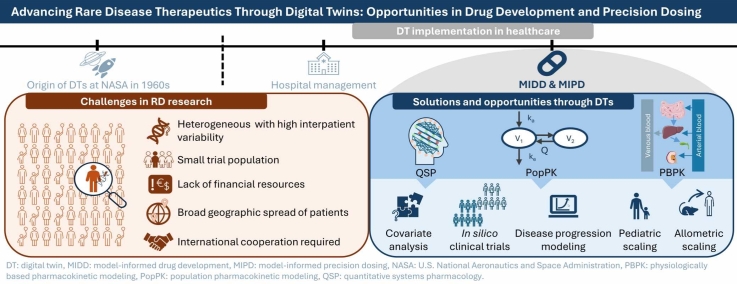

## 1 Background

### 1.1 Digital twins in healthcare

The concept of digital twin(s) (DT/DTs) originated at the U.S. National Aeronautics and Space Administration (NASA) in the 1960s to simulate conditions aboard spacecraft [1]. It has since matured into a core Industry 4.0 technology, denoting a persistent, data-linked relationship between a physical entity and its digital counterpart [2].

**Fig. 1:**
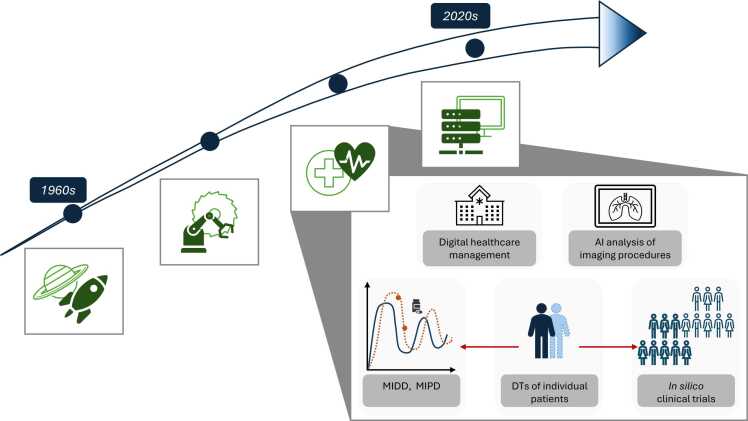
Evolution of digital twin technology. From the 1960s to the present with emphasis on their growing potential in healthcare. Originating in engineering, DTs have been adapted for healthcare applications including hospital management, imaging analysis, drug development, and patient-specific modeling. DTs are embedded in modeling and simulation concepts adopted by regulatory agencies to support clinical decision-making through MIDD and MIPD. Combined with advances in AI and ML, DTs provide new state-of-the-art opportunities in healthcare research such as *in silico* clinical trials and personalized treatment strategies. AI: artificial intelligence, DT: digital twin, MIDD: model-informed drug development, MIPD: model-informed precision dosing [1], [4].

In the context of healthcare, DTs can create dynamic computational representations of human biology supporting drug development and disease management. Following Bruynseels et al., when applied to humans, DTs can be conceptualized as *in silico* representations of individuals that evolve over time integrating molecular, physiological, and lifestyle data [3]. Combined with machine learning (ML), artificial intelligence (AI), and advanced computational simulation techniques, DTs enable the prediction of disease trajectories and therapeutic responses with the ability to extrapolate beyond observed data [4]. The evolution of the DT concept, highlighting its transition from industrial origins to healthcare applications, is depicted in Fig. 1.

**Fig. 2:**
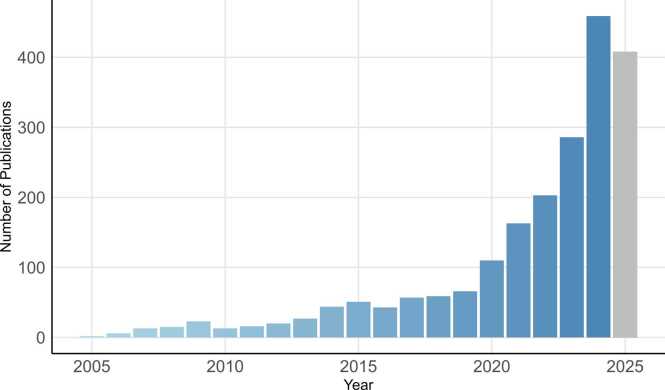
Publications of digital twins in healthcare. Number of published journal articles in PubMed focusing on DTs in healthcare research between 2005 and 2025. For 2025, research articles published before July 24th, 2025 (date of this search) were included. Search terms in PubMed are listed in Table S1 of the Supplemental Materials.

The number of publications on DTs in healthcare research has grown rapidly over the past 20 years (see Fig. 2), reflecting growing interest in DT-enabled drug development, patient management, and treatment optimization.

DTs are currently being developed and implemented for non-communicable diseases such as diabetes mellitus, cardiovascular diseases, mental health disorders, Alzheimer´s disease, and with a strong focus on oncology [5], [6].

In a 2023 review, Chu et al. highlighted the application of DTs in diabetes management and identified four key features that make DTs valuable assets in patient care: (i) integration of diverse data sources, (ii) simulation of physiological processes, such as organ function, pharmacokinetics and pharmacodynamics, tailored to the intended use, (iii) generation of patient-specific treatment recommendations, and (iv) continuous integration of real-time data to account for physiological changes over time [6].

These capabilities may help reduce treatment costs and strengthen prevention strategies [6]. Consistent with the Gartner Hype Cycle, DTs are projected to achieve practical applicability within the next few years [7].

### 1.2 Challenges in rare disease research

In 2024, Gen Li highlighted the growing pressures on clinical research, including increasing trial complexity and demands for cost-effectiveness [8]. DTs may help mitigate these pressures by enabling sponsors to better understand and align with the target population before trial initiation, thereby reducing costly protocol amendments and demonstrating market traction in trials [8].

Rare disease(s) (RD/RDs) trials face these general challenges as well as domain-specific barriers. In the United States, the 1983 Orphan Drug Act defines an RD as a condition affecting fewer than 200,000 people or not expected to generate sufficient revenue to cover development costs [9]. In the European Union, RDs are defined as those affecting fewer than 5 in 10,000 individuals [10]. To date, approximately 7000 distinct RDs have been identified, collectively affecting around 6 % of the global population, equivalent to several hundred million people worldwide [10], [11].

**Fig. 3:**
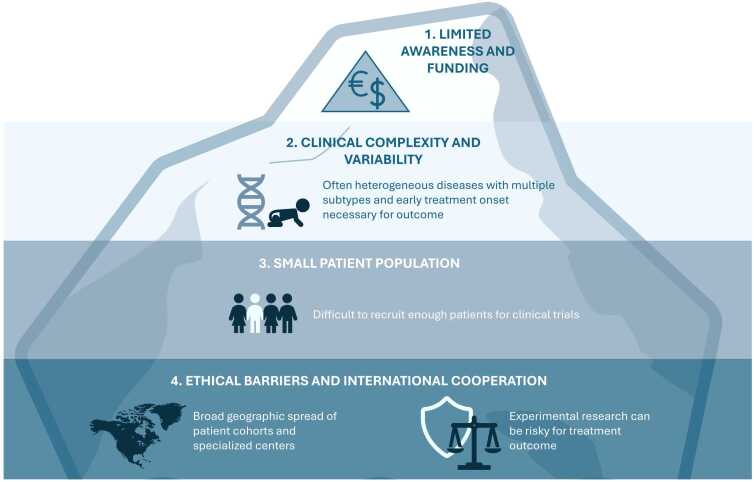
Challenges in rare disease research and treatment strategies. Challenges in RDs mean treatments often remain symptomatic. Even when available, therapies are typically costly and inaccessible, particularly in resource-limited settings. Physicians frequently depend on symptomatic care and trial-and-error approaches, while disease progression remains hard to predict. RDs: rare diseases [12], [14], [16].

Although RDs are highly heterogeneous in etiology, clinical presentation and prognosis, they share common characteristics. Many are chronic, non-preventable, often life-threatening, and frequently driven by genetic or environmental causes. Key barriers to RD drug development and successful trials include (see Fig. 3): (i) delayed diagnosis, (ii) limited understanding of disease mechanisms, (iii) small and geographically dispersed patient populations, (iv) limited data availability requiring broad international collaboration, (v) insufficient natural-history knowledge, and (vi) constrained trial funding [12]. These challenges add to the general difficulties already described for clinical trials and create significant barriers to treatment development and trial execution in RDs [12]. One strategy to address some of these challenges is drug repurposing, which involves identifying new therapeutic uses for drugs that have already been approved for other conditions to accelerate development timelines and reduce costs [13]. This approach is particularly valuable in the field of RDs, as the efficacy and safety profile of repurposed drugs is already known in other populations [13]. Since RD patients are often considered a particularly sensitive population, this can significantly reduce the burden of complex traditional clinical trials [13].

In 2019, Yang et al., analyzed more than 350 RDs and estimated their combined economic burden at $997 billion (USD) [14]. Diagnostic delays can range from six months to twenty years, as reported by Phillips et al., with such delays diminishing the effectiveness of therapies that require early intervention [15].

Digital twinning in clinically well-characterized diseases such as diabetes, as investigated by Chu et al., differs markedly from its application in data-scarce RDs such as rare cancer types, as described by Ståhlberg et al. [5], [6]. Both, however, face similar challenges due to patient heterogeneity. In common diseases such as diabetes, disease information is easily accessible because standardized and well-established monitoring methods, such as continuous blood glucose measurement, enable straightforward and frequent tracking of disease progression [6]. Moreover, the well-understood pathophysiology of most common diseases provides researchers with a clear conceptual framework for what a DT should represent and facilitates the collection of descriptive data necessary for modeling. Overall, deep biomedical understanding, strengthened by decades of research, supports the development of more reliable DTs.

By contrast, DTs for RDs such as uveal melanoma or pancreatic cancer are constrained by small cohorts, sparse multimodal datasets, and incomplete understanding of disease mechanisms, forcing researchers to rely on mechanistic simulations or virtual patient populations to fill knowledge gaps [5]. Additionally, the inherent geographical dispersion of RD patients makes recruitment for clinical studies and data acquisition far more difficult than in common conditions. Despite these differences, both domains must account for extensive biological variability. This is presented, for instance, by the diabetic twins, which adapt dynamically to lifestyle and metabolic fluctuations, and by the cancer twins, which model tumor evolution and therapy response across diverse individuals [5], [6].

### 1.3 Regulatory perspective on DT development

The U.S. Food and Drug Administration (FDA) launched the Critical Path Initiative (CPI) in 2004 to address mounting challenges in clinical research, recognizing that the pathway to drug approval had become increasingly complex, inefficient, and costly [17]. At the same time, sponsors increasingly prioritized programs with higher expected returns, while development for RDs became increasingly challenging [18]. As part of the CPI, the FDA emphasized the potential of computer-based predictive modeling to support translation from laboratory concept to approved medical products [18]. Since then, both the FDA and the European Medicines Agency (EMA) have promoted the application of such methods and published a series of guidance and concept papers that define good practice in modeling and simulation. These include recommendations on physiologically based pharmacokinetic (PBPK) and population pharmacokinetic (PopPK) analyses, reflections on the use of AI/ML in drug development, best practices for model-informed drug development (MIDD), strategic AI work plans, and joint initiatives under the International Council for Harmonisation of Technical Requirements for Pharmaceuticals for Human Use (ICH) framework [19], [20], [21], [22], [23], [24], [25], [26], [27].

In their guidelines, FDA and EMA use a risk- and context-of-use–based standard for model credibility. For PBPK and PopPK, sponsors are expected to provide transparent model development and assumptions, validation against observed data, predictive-performance diagnostics, and a clear statement of model uncertainty and limitations, with enough detail for reproducibility [19], [20], [25], [26]. In the context of drug manufacturing, AI/ML validation is integrated into established quality systems and governed by current Good Manufacturing Practice (cGMP) requirements. Sponsors are expected to define the intended use and performance metrics, validate models with representative data, ensure data integrity, control model updates (e.g., retraining), and continuously monitor operational performance. Both agencies encourage early engagement with regulators [21], [22], [23], [24], [27], [28]. The modeling and simulation approaches differ in their use in regulatory decision making and are therefore addressed separately in submissions. PBPK and PopPK models provide model-informed evidence (MIE), meaning they generate evidence from mechanistic or statistical models and are evaluated against observed data with diagnostics and uncertainty analyses matched to the model’s intended use. By contrast, decision-support-systems (DSS) are software systems that generate recommendations or automate controls affecting development or manufacturing decisions (e.g. dosing aids). In submissions, MIE is provided as analysis reports showing model development and assumptions, validation (e.g., goodness-of-fit plots, visual predictive checks), sensitivity, uncertainty, and clear linkage to the decision context [19], [20], [25], [26]. DSS are assessed under cGMP expectations with lifecycle validation to the stated context of use, documented data integrity, change control for updates or retraining, and ongoing performance monitoring [29].

To date, regulatory agencies have not issued guidance specifically tailored to the use of DTs within the MIDD paradigm. Nevertheless, both the FDA and EMA have begun exploring the integration of DTs into regulatory processes. For example, in its Center for Drug Evaluation and Research’s AI discussion paper, the FDA describes DTs as *“an emerging method that could potentially be used in clinical research”* [21]. Similarly, the EMA has announced plans to conduct a *“technical deep dive, looking in detail at specific tools and techniques (e.g., digital twins)”* over periods of up to six months in both 2024 and 2026 in its multi-annual AI workplan [23]. While these actions signal growing regulatory interest, a DT-specific framework has yet to be established and is expected to continue evolving over the coming years.

### 1.4 Modeling framework of DT development

DT development in RD research can be supported by various modeling tools. Pharmacometric analyses apply mathematical and statistical models, which are broadly categorized into pharmacokinetic (PK) and pharmacodynamic (PD) models [30]. PK describes the behavior of a drug within an organism, covering liberation, absorption, distribution, metabolism, and elimination (LADME) [30]. PD describes the effect of a drug observed at a given concentration [31].

**Fig. 4:**
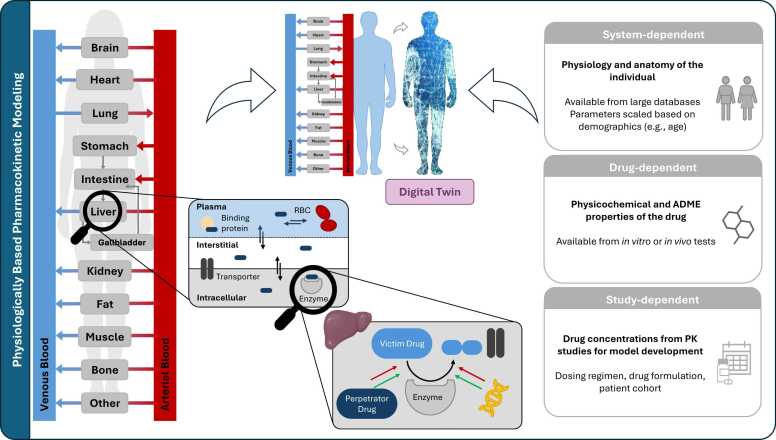
Concepts of physiologically based pharmacokinetic modeling. PBPK models represent the body as interconnected compartments with physiological resemblance, allowing description of processes from the organ to the cellular and subcellular level. Input parameters shown on the right include (i) system-dependent, (ii) drug-dependent and (iii) study-dependent parameters. PBPK models build a robust foundation for patient-specific DTs by incorporating individual patient-dependent data. ADME: absorption, distribution, metabolism and excretion, e.g.: example given, PBPK: physiologically based pharmacokinetic modeling, PK: pharmacokinetic, RBC: red blood cells. Drawings by Servier, licensed under CC BY 3.0 [30], [32], [35]

Here, PBPK is a well-established approach, in which the body is divided into compartments representing organs and tissues linked by blood flow (see Fig. 4) [30], [32]. Each compartment can be further divided into plasma, interstitial, and intracellular sub-compartments, allowing for mechanistic investigation at cellular and subcellular levels [32].

PBPK models support diverse applications in drug development and translation. They enable the mechanistic investigation of drug-drug interactions (DDIs) by simulating the modulation of metabolizing enzymes or transporters [32], [33]. Moreover, PBPK frameworks enable the generation of virtual populations, i.e., a virtual representation of a clinical study cohort through integrating demographic and physiological characteristics of patients [33]. PBPK models can incorporate species-specific enzyme and transporter expression levels to facilitate *in vitro-in vivo* extrapolation (IVIVE) [34]. With additional species-specific parameters such as body weight or organ volumes, PBPK modeling allows for extrapolation from preclinical (animal) data to humans, and from adults to pediatric populations, often through allometric scaling [34].

These features provide a robust foundation for patient-specific DTs by (i) capturing human anatomy and physiology in detail, (ii) allowing individualization through patient-specific parameters (e.g., age, genetics, or disease state), and (iii) enabling mechanistic "what-if" simulations such as untested DDI scenarios. Thus, PBPK models can form the physiological and pharmacokinetic framework underlying a patient’s DT.

Empirical PK models are typically implemented as population PK (PopPK) models, which can serve as a prior for patient-level predictions. PopPK models are data-driven, typically using nonlinear mixed-effects modeling methods to describe observed data while capturing both population-level trends and inter-individual variability [30]. Despite limited mechanistic interpretability, these models are valuable for individualizing DT components through the identification of potential sources of variability (covariates) [30].

Beyond PK modeling, PD modeling addresses the clinical effects of drugs. PK/PD models mathematically link drug concentrations (through PK models) to their pharmacological effects (e.g., biomarker changes) to describe a dose-exposure-response relationship and clinical outcomes [31]. This is done, for example, in early drug development, where PD models often capture simple exposure-response relationships (e.g. IC50, E_max_). Outside of classic PK/PD modeling, physiome models are whole-body, multi-organ physiology frameworks that explain and simulate integrated physiology and pathophysiology using a biophysically based approach, often modeling anatomy and spatial gradients, rather than relying solely on the aggregated compartmental structures typical of PBPK or PopPK models. Mechanistic equations (often ordinary differential equations) connect submodels which can be combined to support DT development. In this field, the International Union of Physiological Sciences (IUPS) Physiome Project and the Virtual Physiological Human (VPH) initiative are leading projects with the goal to “*capture all these fragments of knowledge into predictive and personalised models that will make possible the investigation of the human body as a whole”* [36]. The integration of drug PK into a physiome-based PD model is the basic concept of quantitative systems pharmacology (QSP) modeling. It extends PD concepts to biological networks and can mechanistically capture drug-disease biomarker interactions [31].

These approaches are complementary: PBPK models quantitatively describe LADME processes; QSP and PK/PD models characterize drug-target interactions and downstream effects; and empirical PK and PD models identify sources of variability in drug exposure and response. Together they can provide a foundation for personalized, mechanistic, and population-informed DTs. The methodological basis of each modeling approach alone is already well established and available for broader, complementary implementation.

In addition, these modeling techniques can be used to perform *in silico* clinical trials that can simulate clinical scenarios and predict the safety, efficacy, and outcomes of medical interventions, consequently reducing reliance on animal studies and early-phase human trials [37]. Within this context, DTs can serve as virtual control arms, extending predictions beyond clinical trial data and supporting assessment of efficacy and safety endpoints [8].

**Fig. 5:**
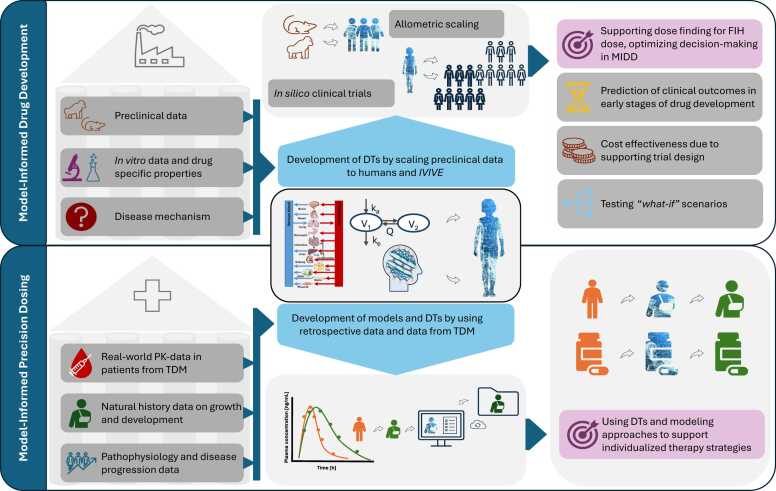
Implementation of digital twins in model-informed drug development and model-informed precision dosing. DTs extend the capabilities of both MIDD and MIPD through integration of patient-specific modeling and data. In MIDD, they use preclinical, *in vitro*, and mechanistic disease data to create virtual cohorts, enabling smaller trials, variability prediction, and informed development decisions. In MIPD, real-world patient data underpins models that simulate drug PK/PD in real time, allowing dynamic dose adjustments. DTs: digital twins, FIH: first-in-human dosing, *IVIVE*: *in-vitro-in-vivo* extrapolation, PK: pharmacokinetic, MIDD: model-informed drug development, MIPD: model-informed precision dosing, TDM: therapeutic drug monitoring. Drawings by Servier, licensed under CC BY 3.0 [8], [24], [35]

The modeling approaches described above form the basis for DT development within MIDD and model-informed precision dosing (MIPD). Both concepts are increasingly applied across the drug life cycle, from clinical trial design to bedside dosing, and are reinforced by regulatory expectations. For example, Lee et al. reported a six-fold increase (2000–2008) in FDA submissions incorporating pharmacometric analyses [38]. MIDD has been defined by Marshall et al. as a “*quantitative framework for prediction and extrapolation, centered on knowledge […] generated from integrated models […] and aimed at the quality, efficiency and cost effectiveness of decision making*” [24]. MIPD becomes particularly relevant after drug approval, where it supports individualized dosing by incorporating patient-specific characteristics such as age, weight, organ function, genotype, and disease state. Fig. 5 illustrates the modeling toolkit used in MIDD and MIPD and the enhancement of both through DT employment.

These approaches are already applied in RD research to compare novel treatments with standard of care and to explore pathophysiological heterogeneity among patients [39], [40], [41]. In oncology, where several tumor types qualify as RDs, the use of virtual control arms shows promise for eliminating the need for placebo groups when patient numbers are limited [42]. Nevertheless, this literature review indicated that, despite achieving high biological fidelity, current RD modeling often falls short of representing a full DT, especially when compared with implementations in more common diseases such as diabetes mellitus [6]. To clearly distinguish a true DT from models that improve disease understanding or predict therapy outcomes but lack individual-patient resemblance and the essential “twinning” aspect, the term DT is defined below before reviewing current research.

### 1.5 DTs in drug development and application

In this review, the term ‘DT’ for drug application and development refers to a dynamic, continuously updateable computational representation of an individual patient. It integrates mechanistic models (e.g., PBPK, QSP), empirical models (e.g., PK/PD), and population-based statistical models (e.g., PopPK, PopPK/PD) with patient-specific and real-world data to predict clinically relevant outcomes and support decision-making across MIDD and MIPD.

DTs fuse heterogeneous data sources (clinical records, trial data, -omics, imaging, and sensor/wearable streams) to describe physiological processes, disease trajectories, and treatment effects, including organ-level responses, metabolic and transporter-mediated interactions, and immune dynamics. Calibrated to an individual’s characteristics (demographics, physiology, genotype, comorbidities, concomitant medications), the twin is iteratively updated as new data accrue to maintain close alignment with the patient (“twinning”). An essential aspect of DTs is their adaptability, allowing the *in silico* representation to be calibrated to an individual patient, capturing key personal traits and characteristics.

### 1.6 Scope of the review

This review evaluates the potential of current DT technologies to advance therapeutics in RDs. Here, we focus on enabling and augmenting clinical trials (e.g., trial design, enrichment strategies, and virtual control arms), while also considering their value for exemplifying RD pathophysiology. Specifically, we (i) define terminology and summarize the foundations of DTs and their current applications in healthcare with an emphasis on RDs, (ii) reveal RD-specific challenges in research and clinical trials, and (iii) assess how DTs may address these challenges through an example RD use case. The review concludes by outlining validation requirements, data and regulatory considerations, and key knowledge gaps and priorities for future research.

## 2 Literature review

### 2.1 Methodology of literature research

**Fig. 6:**
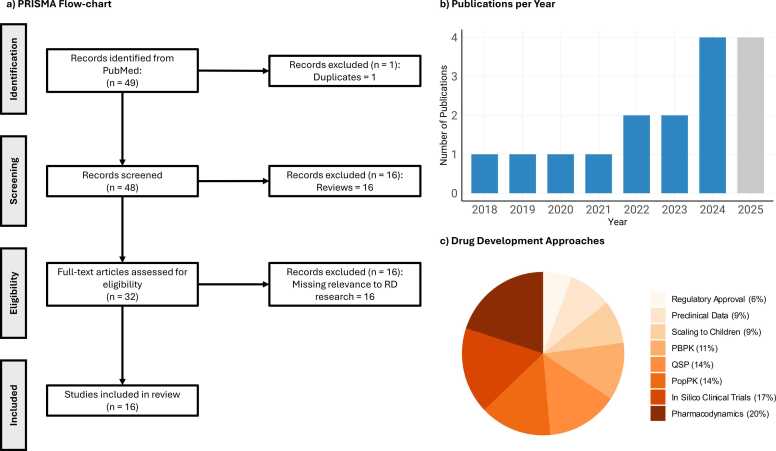
Analysis of eligible research articles. a) PRISMA flow chart of the literature search [43]**.** The chart was generated according to the performed literature search using the PubMed database on July 8th, 2025. b) Number of published original articles given for each year. c) Different approaches and techniques used in the presented models. n: number, PBPK: physiologically based pharmacokinetic modeling, PopPK: population pharmacokinetic modeling, PRISMA: Preferred Reporting Items for Systematic reviews and Meta-Analyses, QSP: quantitative system pharmacology, RD: rare disease.

A comprehensive literature search on the topic of DTs in RDs was conducted using PubMed. The search terms and additional filter criteria used for the literature search are listed in Table 1. Results were screened according to criteria presented in Fig. 6, alongside a detailed quantitative evaluation of the articles included. Only original research articles were considered for inclusion, and records were excluded when no connection to RDs was presented, or full text was unavailable. The search yielded 49 records. After removing duplicates and applying prespecified exclusion criteria such as non-original publications (e.g., reviews), and studies without a RD focus (e.g., communicable diseases, common cancers, or prevalent disorders such as Alzheimer’s disease), in total, this review included 16 studies on RDs that reported the use of DTs and/or QSP, PBPK, PopPK, PK/PD models, and AI/ML meeting all inclusion criteria. Details on the included studies can be found in Table S2 of Section S2.1 of the Supplement.

**Table 1:** Literature search term and additional filter criteria using the PubMed database (search performed on 8th of July 2025).

**Nr.**	**Search term**			**Additional criteria**
1	(("physiologically based"[tiab]) OR ("pharmacokinetic model"[tiab]) OR ("pharmacodynamic model"[tiab]) OR ("pk/pd model"[tiab]) OR ("pk model"[tiab]) OR ("pop-pk"[tiab]) OR ("poppk"[tiab]) OR ("pbpk"[tiab]) OR ("qsp"[tiab]) OR ("quantitative systems pharmaco*"[tiab]) OR ("in silico clinical"[tiab]) OR ("digital twin"[tiab]) OR ("virtual twin"[tiab]) OR ("model*informed"[tiab]))	AND	(("rare disease"[tiab]) OR ("orphan disease"[tiab]))	Language = English, only full text available articles, only original research
2	(("physiologically based"[tiab]) OR ("pharmacokinetic model"[tiab]) OR ("pharmacodynamic model"[tiab]) OR ("pk/pd model"[tiab]) OR ("pk model"[tiab]) OR ("pop-pk"[tiab]) OR ("poppk"[tiab]) OR ("pbpk"[tiab]) OR ("qsp"[tiab]) OR ("quantitative systems pharmaco*"[tiab]) OR ("in silico clinical"[tiab]) OR ("orphan disease"[tiab]) OR ("rare disease"[tiab]) OR ("model*informed"[tiab]))	AND	(("virtual twin"[tiab]) OR ("digital twin"[tiab]))	Language = English, only full text available articles, only original research
[tiab]: search in title and abstract

The 16 included articles were thematically divided into the following six chapters disease progression modeling (2.2), scaling from preclinical data to first-in-human dosing (2.3), scaling from adults to children (2.4), modeling in scarce data scenarios (2.5), drug interaction modeling (2.6) and clinical accessibility to physicians and patients (2.7), as well as a summarizing subchapter (2.8) on the evaluation of DT implementation in the field of RDs and their alignment to the DT definition provided in this review.

In each chapter, representative studies are highlighted to showcase state-of-the-art modeling approaches and the potential of DTs in addressing RD-specific challenges. An overview of the included studies can be found in Table S3 of Section S2.2 of the Supplement with details on model type, data source, validation method and key outcome.

The presented review provides a comprehensive overview of the current state of research in the context of modeling and digital approaches in RDs. Compared to the reviews identified in the PubMed search described in 2.1, our work systematically screened the available literature (see Table S4 of Section S2.3 of the Supplement). To the best of our knowledge, no comparable review has been published to date. Existing reviews in this field mostly address broader applications of modeling techniques and AI in common diseases such as cardiovascular and Alzheimer’s disease or summarize insights from specific workshops related to QSP in RD drug development [44], [45], [46]. However, none of these studies systematically assess the literature on DT and model-based approaches in RDs as comprehensively as the present review.

### 2.2 Disease progression modeling

To account for heterogeneity in disease severity between patients of the same disease, Yıldırım et al. conducted an *in silico* modeling study. The study aimed at improving clinical decision-making for patients with pulmonary atresia with intact ventricular septum, a rare congenital heart condition where treatment largely depends on the size and function of the right ventricle (RV). While patients with clearly underdeveloped or well-developed RVs typically receive single- or biventricular repair, those with in-between RV size categories pose a significant clinical challenge due to the uncertainty surrounding optimal surgical strategy. To address this gap, the authors developed a computational lumped parameter model of the cardiovascular system and created DTs representing virtual patients across a range of RV sizes. These DTs mimic real-world hemodynamics by incorporating variability in physiological parameters, enabling simulation of surgical outcomes after different repair strategies. The authors differentiate between biventricular and one-and-half ventricle (1.5 V) repair. Besides patients with too low indexed pulmonary flow, remaining patients showed an increase in oxygen saturation. Furthermore, mean aortic pressure increased by 43 % after biventricular repair and 59 % after 1.5 V repair. The model predicted that 30 % of digital patients did not achieve adequate blood flow and oxygen saturation after biventricular repair and were therefore considered more suited for 1.5 V repair. About 14 % of these reclassified patients were predicted to fail both repair strategies and would require another surgery. Another finding was that patients with an RV size greater than 22 mL/m² had a high probability of successful biventricular repair. This *in silico* approach demonstrates how computational modeling and DTs can complement clinical decision-making in congenital heart disease. By simulating individual hemodynamic responses based on patient anatomy, the model enables a more data-driven, individualized treatment strategy, especially for complex or ambiguous cases where empirical evidence is scarce [47].

Kaddi et al. developed a QSP and DT framework, informed by the well-characterized pathophysiology of infantile-onset Pompe disease (IOPD) to support its treatment evaluation. IOPD is a rare, life-threatening, and highly heterogeneous disorder characterized by tissue glycogen accumulation and elevated hexose tetrasaccharide (Hex4) biomarker levels caused by acid α-glucosidase deficiency. The standard of care, enzyme replacement therapy (ERT) with alglucosidase alfa, shows variable patient response due to differences in disease onset, progression, and tissue involvement. To address these challenges, the authors developed a mechanistic QSP model that simulates glycogen dynamics by integrating molecular pathways across key tissues. This model served as the foundation for generating DTs which were replications of patients from the Mini-COMET trial. The trial compared the effects of alglucosidase alfa and a next-generation therapy, avalglucosidase alfa, in IOPD patients who had shown declining response to standard ERT. Since IOPD and late-onset Pompe disease (LOPD) are caused by the same gene and share a similar pathophysiology, patient data from both diseases were included for this study. The model reflects the differences between IOPD and LOPD based on the degree of acid α-glucosidase deficiency. First, the pathways of glycogen in healthy patients were simulated successfully. After adapting the base model to the acid α-glucosidase deficiency in LOPD patients, it successfully captured the accumulation of glycogen in muscle and other tissues, as well as the corresponding urine Hex4 levels. For creating DTs of each LOPD patient, observed urine Hex4 at baseline and demographic data were used to calculate organ volumes and urine creatinine levels of each patient of the COMET trial. Combining the QSP model with the DTs, the model was successfully confirmed with datasets of both treatment options which were not used for model development. As a final step, the model was refined to reflect the characteristics of pediatric IOPD patients, who have an enzyme activity of less than 2 % and are typically much younger, with lower body weight and smaller organ volumes. The authors then constructed IOPD DTs for patients in the Mini-COMET trial. Using these, they simulated a virtual population and classified the patients as responders or non-responders [40].

In total, three dosing regimens were used as *in silico* trial: (i) alglucosidase alfa monotherapy, (ii) combined therapy of alglucosidase alfa followed by a switch to avalglucosidase alfa, and (iii) avalglucosidase alfa monotherapy. All three dosing regimens were simulated for each DT. Simulation results showed a greater reduction in urine Hex4 with the new treatment avalglucosidase alfa, consistent with more effective glycogen clearance. This study is a prime example of how DTs integrating QSP models can contribute to a better understanding of pathophysiology and disease progression. It can help to determine the first-in-human dose and compare different treatment options despite the limited data available [40].

### 2.3 Scaling from preclinical data to first-in-human dosing

Several studies have demonstrated the possibilities of scaling animal data to humans even in new therapy approaches in preclinical settings. Desai et al. demonstrated a special application of utilizing a QSP model to describe the dose-response relationship in Clustered Regularly Interspaced Short Palindromic Repeats (CRISPR-Cas) therapies, scaling from mice and non-human primate to humans. The objective was to implement the novel therapy for the treatment of transthyretin amyloidosis (TA), a RD in which the protein transthyretin (TTR) is formed incorrectly, leading to deposits in organs. The primary goal of the therapy is to prevent the formation of TTR and consequently slow down disease progression. Using the model, the authors demonstrated the feasibility of predicting the distribution of the pharmaceutical agent’s key components within the body and estimating the inhibition of TTR formation. Accounting for variability, multiple administration strategies were simulated. Additionally, the authors modeled and scaled physiological processes across virtual species, with the potential to incorporate individual patient characteristics, thus completing the DT approach. As a result, the model enabled a more accurate estimation of the first-in-human dose and ultimately enhanced understanding of the dose-response relationship [48].

Describing disease mechanisms, Nguyen et al. developed a mechanistic, multiscale model to estimate the human efficacious dose of FS-EEE-Fc, an engineered follistatin fusion protein, for the treatment of Duchenne Muscular Dystrophy without access to human PK data. It integrates the biological interactions between FS-EEE-Fc and key molecular targets such as myostatin and activin A, both negative regulators of muscle growth. By inhibiting these targets, FS-EEE-Fc is expected to enhance muscle mass. Instead of using human PK, the model leveraged preclinical PK/PD data from mice, rats, and monkeys, along with clinical data from other myostatin inhibitors to calibrate and validate the system. Through allometric scaling and detailed modeling of target engagement, receptor occupancy, and downstream pharmacodynamic effects, the model successfully extrapolated preclinical insights to predict first-in-human dose projections. A weekly dosing of 3–5 mg/kg is predicted to reach necessary clinical treatment outcome. The authors provided a web-based tool to use the QSP model to predict FS-EEE-Fc exposure in humans across different doses and regimens. The study represents the precursor of a DT. Here, a more complete DT can be achieved by incorporating individual patient data into the model [49].

A similar approach was used by McBride et al. In their study, they used a QSP model to mechanistically describe congenital thrombotic thrombocytopenic purpura (cTTP), an RD caused by a deficiency of a disintegrin and metalloproteinase with thrombospondin motifs 13 (ADAMTS13). Plasma-based therapy currently replaces ADAMTS13, and recombinant ADAMTS13 (rADAMTS131) has recently been developed. The model simulated the disease by reflecting the interactions between ADAMTS13, von Willebrand factor (VWF), platelets, as well as the binding inhibitors thrombospondin protein 1 and extracellular hemoglobin. The advantages of the new rADAMTS13 therapy using different dosing regimens were demonstrated, and the level of active ADAMTS13 was estimated by the model. Additionally, covariates were discovered that correspond to the actual disease mechanism. The model was used to compare the risk of thrombocytopenia during plasma-based therapy versus rADAMTS13 treatment. With bi-weekly administration, the model predicted a 53 % reduction in the risk of moderate thrombocytopenia and a 59 % reduction in the risk of severe thrombocytopenia. It successfully simulated the pathophysiology of cTTP by generating a virtual population with baseline characteristics based on a Phase 3 cTTP clinical study. The modeling approach was part of the submitted approval application to the FDA, and FDA accepted the results from the QSP model as confirmative evidence proving the advantages of rADAMTS13 compared to plasma-based therapy. This example demonstrates how QSP models can supplement the information required for new drug approval submissions to regulatory agencies. The lack of human data is addressed through the models’ ability to extrapolate from known to unknown scenarios [50].

Using QSP modeling as a foundation for DTs, Susilo et al. investigated the dose-exposure-response relationship of mosunetuzumab in patients with non-Hodgkin’s lymphoma (NHL), comparing aggressive and indolent subtypes. Although NHL represents a relatively prevalent group of hematologic malignancies, individual subtypes such as diffuse large B-cell lymphoma (DLBCL, aggressive) and follicular lymphoma (FL, indolent) are classified as RDs [51]. These diseases can arise from either T- or B-cells. Mosunetuzumab is an IgG-bispecific antibody that simultaneously binds to CD3 receptors on T-cells and CD20 receptors on B-cells, redirecting endogenous T-cells to eliminate malignant B-cells. This mechanism of action was implemented in a QSP base model, which was then individualized for each patient enrolled in a phase I/II trial. To account for biological, pharmacological, and tumor-related variability, the authors generated multiple DTs with varying parameter sets. These virtual populations were evaluated, and the 25 DTs with the lowest objective function values, indicating the best fit to clinical data were selected for additional simulation studies. Susilo et al. explicitly built a per-patient, mechanistic replica of each trial participant and used these for individualized “what-if” simulations. The approach substantially meets criteria for a DT by (i) individualizing the QSP model via parameter estimation to reproduce each person’s tumor size trajectory, often with multiple alternate parameterizations per individual that collectively constitute the patient’s twin(s) and the virtual population, (ii) fusing heterogeneous data to construct those twins, and (iii) emphasizing the mechanistic fidelity that enables inference on clinically unmeasured biomarkers and decision support for efficacy as well as safety and dosing strategy [52].

Different dosing regimens were tested for DLBCL and FL patients. The simulations showed that higher mosunetuzumab exposure led to a greater reduction in B-cell counts by day 42. Since many patients had previously received rituximab, which targets the same CD20 epitope, higher doses of mosunetuzumab were required to overcome potential competition for binding sites. The model successfully reproduced dynamic changes in B- and T-cell populations and was consistent with biopsy observations. Moreover, it indicated that T-cell trafficking between blood and tumor tissues should be incorporated to better capture treatment effects [52].

Overall, this study demonstrates a promising DT approach based on mechanistic QSP modeling. The framework could be extended by integrating clinical biomarkers to distinguish responders and non-responders. However, the presented DTs do not incorporate real-time patient data or predict mechanisms of therapeutic resistance in their current state. Despite these limitations, the integration of preclinical, *in vivo*, *in vitro*, and clinical data enabled the development of a robust mechanistic model that serves as a foundation for personalized DTs [52].

### 2.4 Scaling from adults to children

In the context of first-in-children dose estimations, Li et al. developed a PopPK model to extend approval for the use of the drug nexviazyme in the treatment of LOPD in children under the age of 16. Conducting clinical trials in this age group is challenging because the disease is very rare in children, and therefore there are only a few eligible patients available. To address this, the researchers used data from two phase 1/2 trials in patients with LOPD, one phase 3 trial in patients with LOPD and a phase 2 trial in patients with IOPD. In patients with LOPD, age ranged from 16 to 78 years old and in patients with IOPD age ranged from one to eleven years old. A three-compartment model with parallel linear/nonlinear clearances from the central compartment was built, incorporating body weight as a covariate. This model was scaled to children using an exposure-bridging approach. Because LOPD has similar pathophysiology and disease features across ages, pediatric dosing was based on model-informed exposure bridging, supported by IOPD pediatric safety data. Finally, a virtual population of adults with characteristics from clinical trials and a virtual pediatric population younger than 18 years old was generated based on weight-for-age growth charts from Centers for Disease Control and Prevention. With the help of this model, an application for approval for children aged one year and over at a dose of 40 mg/kg could be submitted [53].

Scaling drug pharmacokinetics from adult populations to children is a key application of PBPK modeling. Bonner et al. addressed a key clinical challenge in managing congenital adrenal hyperplasia in children: avoiding both overtreatment, which risks obesity and growth suppression, and undertreatment, which can lead to adrenal crises. To support individualized dosing, they developed a PBPK model of hydrocortisone that accounts for developmental maturation from birth to 18 years. The model successfully described the PK for both immediate- and modified-release formulations, enabling dose optimization across pediatric age groups, ethnicities, and formulations, even in the absence of dedicated clinical trial data. For children between 12 and 18 years, the authors observed a similar PK compared to adults when taking modified-release formulations [54].

Both articles show how the use of PBPK and PopPK modeling for scaling to children represents an important application. Here, the model approaches serve as a tool to extrapolate to special populations where data is often scarce, and clinical trials face ethical and economic challenges. Here, the PBPK and PopPK models serve as a foundation to generate precise DTs by integrating mechanistic processes, scalable organs and organ functions, enzymes and demographic information.

### 2.5 Modeling in scarce data scenarios

Major challenges to DT model development are limited data availability and accessibility. Janssen et al. addressed this by integrating PopPK models with causal inference techniques. By incorporating causal inference, the model could simulate and account for missing or incomplete data more accurately. For example, even if only age data were available, the model could infer other covariates, like weight or factor VIII clearance, based on established causal relationships, leading to reliable predictions of e.g. factor VIII concentrations in hemophilia A patients. This makes it possible to generate realistic virtual patients and replace missing data. Additionally, the generated virtual patients can be dynamically adapted, mirroring the continuous development of a real patient, through the combination of causal inference and the modeling approach. Janssen et al. demonstrated the effectiveness of this approach using hemophilia A as a case study. Their model accounted for both interindividual variability and differences related to the formulation of factor VIII. Importantly, this technique also supports privacy-preserving model development. Since the causal structure is explicitly defined and interpretable, it enables local data integration without needing to centralize or share sensitive patient data. Institutions can use their own protected datasets to train or refine the model locally, while still contributing to overall model improvement [55].

Another example for modeling in scarce data scenarios is described in the study by Carlier et al. An *in silico* clinical trial on virtual patients was performed to explore the therapeutic potential of bone morphogenetic protein (BMP) treatment in children with congenital pseudoarthrosis of the tibia (CPT) which is a RD characterized by progressive bowing and spontaneous fractures of the tibia. Due to FDA restrictions on BMP use in skeletally immature patients, clinical evaluation of its effectiveness in this group is limited. To address this, the authors developed a mechanistic model of murine bone regeneration, coupled with ML, to generate a virtual population of 200 subjects, representing both treated and untreated scenarios. A key contribution of the study was the stratification of virtual subjects based on treatment response. By simulating each subject’s outcome using a complication index, they categorized responses into four groups: responders, non-responders, asymptomatic, and adverse responders. Clustering analysis confirmed these groupings and demonstrated how virtual trials can uncover inter-individual variability in treatment effects [56].

### 2.6 Drug interaction modeling

Polymedication, defined as the use of five or more prescription drugs, is often required in the management of RDs, as these conditions frequently affect multiple organ systems [57]. As a result, DDIs may occur and represent a major cause of adverse drug reactions, which significantly contribute to hospital admissions and in-hospital mortality [58], [59]. As stated in chapter 1.4, mechanistic approaches such as PBPK modeling are effective tools to model DDIs. The PBPK models presented here focus on predicting the DDI potential of drugs used in an RD context, without explicitly incorporating RD-specific pathophysiology. They are not considered DTs in the author´s sense (the reader is referred to chapter 1.5 for the exact definition); however, they provide a foundation for understanding drug interaction potential, an important consideration for RD patients.

There is a pronounced lack of DDI studies on orphan drugs utilized in RDs compared to drugs used to treat common diseases. To address the challenge of data scarcity, Lee et al. and Sahasrabudhe et al. employed DDI modeling in place of additional clinical studies of vatiquinone and eliglustat to estimate the effect of DDIs on the PK of drugs used in patients with two different RDs [60], [61].

Vatiquinone is used in the treatment of Friedreich’s ataxia, a progressive neurodegenerative disease. Lee et al. employed clinical DDI study data involving the strong cytochrome P450 (CYP) 3A4 inhibitor itraconazole and strong CYP3A4 inducer rifampicin for model development and evaluation and subsequently predicted the effect of moderate CYP3A4 inhibitors fluconazole and moderate CYP3A4 inducer efavirenz on the PK of vatiquinone. Additionally, the effect of vatiquinone on the PK of the sensitive CYP3A4 substrate midazolam and the sensitive CYP1A2 substrate caffeine was predicted despite missing data in patients. Simulating the DDIs with midazolam and caffeine, no significant effect was detectable with CYP3A4 or CYP1A2 substrates. When evaluating vatiquinone as a victim drug, coadministration with fluconazole was predicted to increase AUC by less than 50 %, whereas coadministration with efavirenz was predicted to decrease AUC by about 20 %. Overall, the analysis indicates a minor interaction risk for coadministration with CYP3A4 and CYP2A1 substrates, whereas the model predicts moderate change in the PK of vatiquinone when co-administered with CYP3A4 perpetrators. This allows to initially assess the risk of DDI-related side effects, even without clinical data in humans, which is an important step in ensuring the safety of RD patients with polymedication [60].

Sahasrabudhe et al. developed a minimal PBPK model of eliglustat, a drug used to treat Gaucher disease. Eliglustat is primarily metabolized by CYP2D6 and, to a lesser extent, by CYP3A4. Consequently, eliglustat PK is sensitive to a patient’s CYP2D6 genotype and DDIs with CYP2D6 or CYP3A4 inhibitors. To assess the appropriateness of FDA-recommended eliglustat doses in drug-drug-gene interaction (DDGI) scenarios, the authors simulated the combined effect of CYP2D6 drug-gene interactions (DGIs) and DDIs with the weak CYP2D6 and CYP3A4 inhibitor fluvoxamine and the strong CYP3A4 inhibitor ketoconazole in virtual clinical trials. Simulation results indicate that the standard FDA-recommended regimen of 84 mg eliglustat twice a day resulted in eliglustat concentrations above the safety threshold (> 250 ng/mL) in 41 % of CYP2D6 intermediate metabolizers (IMs) and 25 % of poor metabolizers (PMs) when ketoconazole was co-administered. Adjusting eliglustat doses to 42 mg or 21 mg administered once daily in these scenarios resulted in 3 % of virtual CYP2D6 IMs and 0 % of PMs displaying eliglustat concentrations above the threshold. Conversely, the authors found that no dose adjustments were necessary for CYP2D6 IMs and PMs when eliglustat was co-administered with fluvoxamine as no virtual participant had eliglustat concentrations above the safety threshold. These findings demonstrate the utility of PBPK modeling in optimizing dosing strategies in complex DDGI scenarios and reducing reliance on additional clinical trials [61].

Machavaram et al. investigated the effect of elevated interleukin-6 (IL-6) levels on the PK of various CYP probe substrates. Their goal was to estimate changes in drug exposure in patient populations with chronic inflammation, such as those with neuromyelitis optica (NMO) or NMO spectrum disorders. First, a PBPK model with focus on patients with rheumatoid arthritis (RA) was built, a disease where elevated IL-6 levels are well investigated. By integrating IL-6-mediated suppression of hepatic enzyme expression into a mechanistic PBPK framework, the authors successfully predicted the altered pharmacokinetics of several CYP substrates (including caffeine, S-warfarin, omeprazole, dextromethorphan, midazolam, and simvastatin) in the RA population. Overall, elevated IL-6 levels resulted in increased exposures to CYP probe substrates (except caffeine), with more than a 2-fold increase predicted for CYP3A probe substrate simvastatin. Besides the influence of IL-6 on CYP expression, the effect of CYP polymorphisms on substrate exposure at varying IL-6 levels was investigated. Additionally, the influence of ethnicity on the PK of CYP substrates in combination with elevated IL-6 levels was simulated. The influence of ethnicity was not significant whereas DDIs and DGIs including CYP2C9, CYP2C19, CYP2D6 and CYP3A4 showed a moderate change with different IL-6 levels. The authors extrapolated the framework to NMO patients, leveraging the shared inflammatory phenotype characterized by elevated IL-6. This study showcases a strategic use of PBPK modeling to bridge knowledge from one inflammatory disease population (RA) to another less-characterized rare disease (NMO) [62].

### 2.7 Clinical accessibility to physicians and patients

Despite various approaches, pharmacometric models are rarely used for real patients in clinical practice. In the study by Chelle et al., a PopPK model was developed to ascertain the optimal dosage and dosage intervals for patients with hypoplasminogenemia. Patients with hypoplasminogenemia show an inability to effectively break down excess fibrin, which can lead to the formation of lesions. This RD is treated with Ryplazim®, which is plasma derived human plasminogen. This modeling approach identified that variability is strongly influenced by fat-free mass. The model is a valuable tool for supporting dose finding in pediatric patients and PK-guided prophylaxis. Despite intra- and interindividual variability and the small sample size, a robust model has been developed and is available to use as a tool on the Web-Accessible Population Pharmacokinetic Service - Hemophilia (WAPPS-Hemo) platform. This approach addresses the problem of insufficient data because it allows the incorporation of various PK data from worldwide patients into the model via the platform. Additionally, easy access to the platform facilitates the practical implementation of the models into the real world [63].

### 2.8 Evaluating alignment with DT principles

In general, all assessed manuscripts do not align with the DT definition stated in chapter 1.5 because they lack more than one of the following essential elements we distinguish for a true DT (vs. a purely mechanistic, empirical, or statistical model): (i) representation of an individual patient, (ii) fusion of heterogeneous data sources, and (iii) iterative updating via real-time data transfer, however, their modeling techniques lay much of the groundwork for actual DTs. In many cases only a few steps are needed, for example, Chelle et al. developed a robust PopPK model to guide dosing intervals in hypoplasminogenemia and enabled data flow through the WAPPS-Hemo platform, though the backbone remains a PopPK model derived from just 16 participants.

However, none of the papers implement a mechanism to iteratively update an individual’s twin as new data arrives, which represents a central feature in our DT definition. The authors rely on time-invariant parameters or describe one-time calibrations or cohort simulations rather than an online updating workflow. The presented publications serve not as a fully featured DT, but have the potential to expand their precursor of a DT to a complete DT.

Of all 16 full-text assessments, three of the published articles meet most of the named requirements of a DT for RDs stated in this review in chapter 1.5. These works closely approximate a DT for three reasons. (i) Susilo et al. and Kaddi et al. calibrate the twin to individual characteristics by, for example, estimating parameters per clinical patient, matching demographics of individual patients and selecting individualized virtual patients to match disease trajectory, whereas Yıldırım et al. do not meet the DT criterion of this review here because they model cohort-level virtual patients grouped by RV size rather than per-patient twins. (ii) They fuse heterogeneous data to construct the model: Susilo et al. integrate mechanistic physiology, PK/PD, preclinical cynomolgus monkeys and mosunetuzumab *in vitro* data together with patient-level trial data to generate per-patient twins, Kaddi et al. blend multiple clinical cohorts registry patients and validation datasets into a QSP/PBPK-linked framework, and Yıldırım et al. generate virtual patients from statistical distributions and validate surgical scenarios against published and retrospective. (iii) The challenging key feature of enabling real-time DT updates is not addressed in any of the publications. Finally, although all three examples demonstrate adaptability for “what-if” decision support, they do not fully satisfy the criteria of a DT as defined in chapter 1.5 [40], [47], [52].

Taken together, and as we outline in chapter 1.2, current RD DT implementations lag those in common diseases, for reasons such as practical difficulty of embedding continuous, clinically tractable measurement loops (there is no RD analogue to simple progression tracking like HbA1c in diabetes).

## 3 Use Case: DTs of patients with epidermolysis bullosa

This review discussed various modeling techniques that can support the development of DTs in RD research. These models vary in their level of complexity, some represent entire individuals, while others focus on specific organs, biological processes, or disease mechanisms. Despite these differences, they have demonstrated utility in early-stage clinical development, including first-in-human dose estimations and scaling from preclinical to human data, as well as scaling from adult to pediatric populations. Despite their complementary strengths as described in chapter 1.4, combined modeling approaches are still rarely published.

To maximize the potential of DTs, a modular, building-block approach is proposed as a guide for developing DTs tailored to RDs. This concept is further illustrated using the example of the RD epidermolysis bullosa (EB).

### 3.1 Epidermolysis bullosa

EB describes a group of rare disorders characterized by skin fragility, resulting in blistering and scarring of skin and mucus membranes [64]. There are four main subtypes of EB, each caused by different genetic mutations of essential components of the dermo-epidermal junction zone: (i) EB simplex, (ii) junctional EB, (iii) recessive dystrophic EB (RDEB) and (iv) Kindler EB [64]. Children carrying this rare condition are colloquially called “butterfly children” referring to the fragility of their skin being comparable to butterfly wings. Depending on the subtype, EB can be a severe and potentially life-threatening condition with a high risk for development of squamous cell carcinoma, high mortality rate and generally reduced life expectancy [64].

Around 500,000 patients are affected worldwide, with specific subtypes such as RDEB being particularly rare (incidence of less than 3 per million people) [65], [66]. EB is currently incurable, and treatment focuses on a symptomatic approach with extensive wound care being first-line therapy.

Vyjuvek® (active ingredient: Beremagene geperpavec) is the first FDA approved topical gene therapy for treatment of recurring wounds in RDEB [67]. However, applications are very expensive with costs of around $630,000 (USD) per patient each year and Vyjuvek® treatment is not available in all countries. Another recent FDA approved treatment option refers to Zevaskyn® (active ingredient: prademagene zamikeracel) [68]. This is the first autologous, cell-based gene therapy for RDEB [68]. The treatment uses autologous *COL7A1* gene-modified cellular sheets that are being transplanted onto RDEB wounds, thus requiring extensive lab work for the sheets to be established and applied [68]. For the above-mentioned reasons, only a very restricted number of patients have access to both treatments [67], [68]. Another EMA and FDA-approved therapy for EB is Filsuvez®, a birch bark extract wound gel. However, several novel therapies are in clinical development, including gene and cell therapy approaches as well as new or repurposed small molecules [69], [70], [71].

**Fig. 7:**
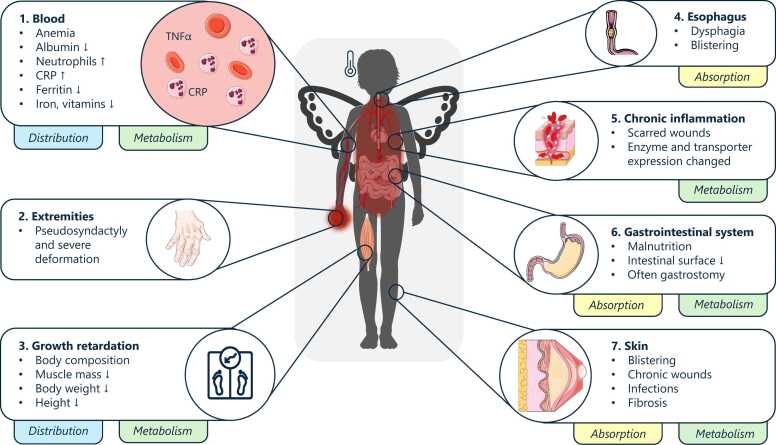
Overview of the unique pathophysiology of recessive dystrophic epidermolysis bullosa patients according to Tang et al. [66]**.** Children with RDEB exhibit changes in various parts of their bodies, which can affect the way drugs are absorbed, distributed, metabolized and excreted. CRP: C-reactive protein, TNFα: tumor necrosis factor α. Drawings by Servier, licensed under CC BY 3.0 [35], [66], [72]

For further detail on the pathophysiology of RDEB, the reader is referred to Fig. 7. Prompt diagnosis shortly after birth is crucial to initiate immediate wound care and overall patient treatment as the disease burden is high and severely impacts child development [66]. Progressive scarring and fibrosis, an essential part of the natural disease history that is potentially aggravated by delayed treatment onset, can lead to limb deformities requiring extensive surgical reconstruction of hands and feet. The ongoing inflammatory processes and involvement of large chronic wounds require a special diet to avoid malnutrition and ensure age-appropriate maturation and growth [66]. In total, patients will receive topical antibiotics, systemic analgesics and additional medication such as proton pump inhibitors, creating a potential risk for DDIs.

Currently, within the EB research field, the use of modeling is commonly limited to *in vitro* or *in vivo* models such as cell samples, three-dimensional organotypic skin models and genetically engineered mice [73]. However, Lalor et al. have created an *in silico* model of mutations in keratin 5 and keratin 14 genes, which cause fatal, severe and generalized EB simplex. The model was successfully used to analyze the strength of protein interactions within the mutated keratin filaments [74]. In contrast to physical models, an *in silico* approach might combine the multiple pathophysiological aspects of EB to drive evidence-based drug development for new effective EB therapies [73].

### 3.2 A guide towards a DT of patients with EB

Building on this knowledge, the potential application of the concepts presented earlier regarding DTs within the RD field should be combined with a DT specifically tailored to the needs of EB patients. A DT of EB patients should incorporate (patho-)physiological features of this heterogeneous patient cohort, considering the type of underlying gene mutations, severity of the subtype as well as individual patient demographics [66]. The first step in constructing a DT for EB patients involves collecting comprehensive patient data. Because EB patients are geographically dispersed, establishing centralized and collaborative data collection systems may address logistical challenges and support comprehensive representation [75]. Moreover, it is crucial that clinicians and researchers publish detailed case reports and share de-identified individual data. Academic institutions are encouraged to make proprietary data accessible for collaborative use, enabling a more complete and diverse data foundation.

Additionally, collecting long-term natural history data is essential to model disease progression. Mellerio et al. were among the first to perform a prospective register study in 2014 (the Prospective Epidermolysis Bullosa Longitudinal Evaluation Study (PEBLES) in London) to collect detailed natural history information about RDEB [76]. Model accuracy depends heavily on capturing and analyzing the longitudinal evolution of clinical, biological, and functional parameters in patients over time. Aside efforts in England, Reimer et al. in Germany investigated the clinical course of growth and anemia in 200 children with EB and published growth charts in correlation to anemia, nutrition and inflammation [77]. However, in both countries, the data were published only as mean values, not as individual data. To better reflect patient variability, data should go beyond aggregated mean values, with more emphasis on publishing individual patient information when possible. Digitization of clinical records and legacy data is also essential to preserve valuable information and make it more easily retrievable for research and modeling purposes. For EB, this involves collecting time-series data from multiple patients on key disease markers, such as wound healing rates, frequency and severity of blistering, skin integrity metrics, pain levels, mobility, and secondary complications (e.g., infections, scarring, or squamous cell carcinoma). Additionally, data needed for model development includes clinical features of each individual patient such as age, gender, weight, height and ethnicity of the affected child alongside blood work such as inflammatory markers (e.g. CRP, TNF-α, TGF-β), hematologic parameters (e.g. serum iron and hemoglobin) as well as disease burden scores (e.g. the Birmingham Epidermolysis Bullosa Severity Score, the Instrument for Scoring Clinical Outcomes of Research for Epidermolysis Bullosa or the Epidermolysis Bullosa Disease Activity and Scarring Index) [78].

**Fig. 8:**
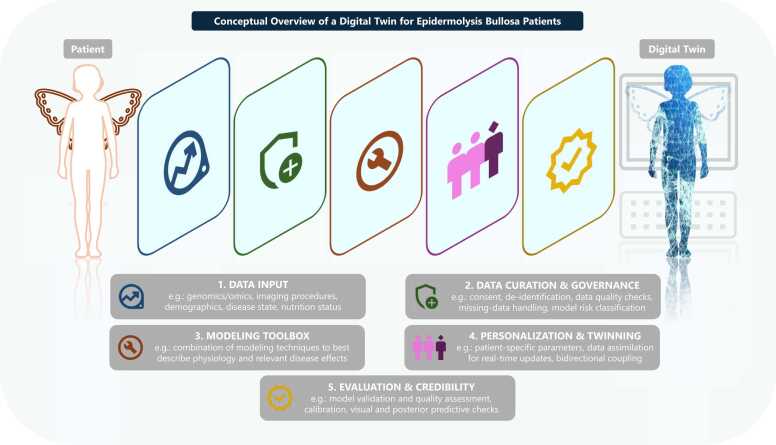
A conceptual digital twin architectural figure. (1) Data and signals include genomics and other omics, clinical images and measurements, patient reported outcomes, and relevant contexts such as infection status, nutrition, and ambient conditions. (2) Curation and governance ensure consent and provenance, interoperability, data quality control, time alignment, handling of missing data, and classification of model risk by context of use and impact. (3) The modeling toolbox combines modeling techniques such as physiome modeling, QSP for mechanistic representation of (patho-)physiology as well as PopPK and PBPK for PK predictions. (4) Personalization and confirmation estimate patient specific parameters, assimilate new data in real time, quantify uncertainty, and confirm predictions through diagnostics including goodness of fit and calibration, visual predictive checks for PK and PD, and prospective checks using, for example, measured exposure. (5) The clinical closed loop delivers twin to care recommendations and explanations, ingests new data from care to twin including images, patient reported outcomes, laboratory results and therapy logs. All of this is repeated during DT development and in clinical implementation and constantly refined. DT: digital twin, EB: epidermolysis bullosa, e.g.: example given. Drawings by Servier, licensed under CC BY 3.0 [35].

Building on the foundation of data availability, the next step includes the selection of modeling approaches that are precisely tailored to the objectives and constraints of the proposed EB DT. Defining the purpose of the DT is essential, whether it is intended for simulating treatment outcomes, predicting disease progression, or supporting personalized therapy, the modeling approaches must be aligned accordingly. A core requirement is the ability of the model to represent individual patient characteristics and capture inter-patient variability, ensuring that the DT reflects the heterogeneous nature of EB. In the case of EB, a promising approach might be to combine drug repurposing with a dual modeling strategy, linking PBPK with PopPK approaches. PBPK modeling can first be applied in healthy adult and pediatric populations, where data are available, to establish a baseline understanding of pharmacokinetics and validate model performance in a well-characterized context. Once validated, the PBPK model can then be adapted to EB by incorporating disease-specific physiological changes. In parallel, PopPK modeling can be used to evaluate these disease-related factors in patient data and uncover and quantify sources of interindividual variability. Together, this dual approach strengthens predictive accuracy and provides a more comprehensive understanding of drug behavior in EB patients. Conceptual details on DT development for EB patients are presented in Fig. 8.

Finally, scientists and clinicians should explore how such a DT model can be made available and used in clinical practice to improve patient care. Here, an online platform where clinicians can input patient-specific demographic and clinical information and subsequently receive estimations on disease trajectory or individualized drug pharmacokinetic predictions are highly desirable.

### 3.3 Ethical considerations

The integration of DTs in RDs, particularly for EB patients, raises several ethical, technical, and clinical challenges that must be addressed thoughtfully. A key concern is ensuring that sensitive health information is de-identified, protecting sensitive patient data [79]. Robust cybersecurity measures are essential to safeguard databases and DTs, ensuring the integrity and confidentiality of patient data. Equally important is the need for transparency with patients regarding how their data will be used. When employing DT technology to simulate disease progression and outcomes, effective data governance is essential to safeguard patient rights and maintain public trust. Data governance refers to the framework of policies, processes, and standards that ensure ethical, secure, and transparent management of data throughout its lifecycle. One crucial component is dynamic consent, a model that allows participants to continuously manage how their data is used through digital interfaces. For example, dynamic consent was carried out in the EnCoRe project in the context of three biobanks at Oxford University enabling patients to modify or withdraw consent for research participation in real time, ensuring flexibility and autonomy [80]. Another key approach is federated learning, which enables algorithms to be trained across multiple datasets held at different institutions without moving the data itself. This preserves privacy while promoting large-scale collaboration, as seen in initiatives like the European Health Data & Evidence Network, which uses federated analytics to health research [81]. Finally, recognizing patient ownership of data strengthens ethical accountability by granting individuals access to, and control over, their personal health information. The MIDATA cooperative in Switzerland, for instance, allows citizens to store and manage their health data securely while deciding who can use it for research purposes [82].

In EB, where data is scarce, it must be considered that using small patient groups to build virtual replicas and extrapolating findings to larger populations can lead to biases and inequalities. For instance, applying a generalized model to specific cases, such as underrepresented ethnic groups with EB, might raise concerns about validity. The question of how patients benefit from contributing their data to DT development must also be addressed. If these technologies generate substantial commercial profit, clear agreements are needed to determine data ownership and ensure that patients are not exploited. DTs of individual EB patients may also reveal distressing insights, such as severe disease progression or a high likelihood of e.g. squamous cell carcinoma metastasis, potentially indicating a shorter life expectancy. In such cases, patients, relatives and caregivers need access to support systems, including psychological counselling and clear, compassionate communication about their prognosis.

For EB patients specifically, the leading EB patient advocacy and support network Dystrophic Epidermolysis Bullosa Research Association (DEBRA), among others, provides easily accessible, clear information about EB and how it can progress. For example, on their “EB Support & Resources” pages, DEBRA United Kingdom (UK) provides dedicated life-stage support, ranging from childhood and adolescence and adulthood phases to end-of-life [83]. Moreover, DEBRA UK’s resources refer people to trusted external services such as Citizens Advice for help with social-care, benefits and rights, ensuring that patients and carers receive holistic support that combines medical, emotional and financial dimensions of living with EB [83]. Beyond EB, non-profit alliances such as European Organisation for Rare Diseases (EURORDIS) offer initiatives like RareConnect, which provide a supportive environment for connection and exchange among patients living with RDs across Europe [84], [85]. The alliance raises awareness, influences health policies and promotes fair access to diagnosis and treatment. Additional examples include specialist care networks like European Reference Networks (ERN)-Skin, part of the European Reference Networks initiative, which link expert centers across Europe to ensure coordinated, high-quality care for rare dermatological conditions, and digital health platforms such as Orphanet, which provide reliable, up-to-date medical information, expert directories, and clinical trial listings to help patients and clinicians make informed decisions [86], [87].

The reliability of DTs is critical, as they may inform life-altering clinical decisions. Developers must ensure the quality and boundaries of these models, demonstrating their ability to predict and inform clinical outcomes accurately. Adopting performance standards, like those used in MIDD by regulatory bodies like the FDA and EMA, could help to establish credibility and efficacy.

## 4 Summary and outlook

RD research faces major challenges including delayed diagnosis, limited knowledge of disease mechanisms, small patient populations, and difficulties in data sharing and availability, all of which challenge treatment development and clinical trial design [12]. Additionally, the high economic burden (e.g., expensive treatments), lack of funding, and need for international collaboration further complicate progress in understanding and managing RDs [12]. To address these challenges, this review focused on the potential of DTs to assist in drug research and development in this specific field.

DTs enable extrapolation from the known to the unknown as seen in the approach by McBride et al. and their successful description of the advantages of rADAMTS131 compared to plasma-based therapy in cTTP despite the lack of available human PK data [50]. The work performed by Nguyen et al. also exemplifies the value of QSP modeling in early-stage drug development, particularly in RD settings where human data is scarce or unavailable [49]. It highlights how mechanistic models can guide dosing strategies and support translational decision-making, even before first-in-human studies begin. The authors recognize the significant potential of this approach in the context of RDs, given the small sample sizes and the valuable insights it provides into disease mechanisms. When evaluating relevant DDI scenarios, PBPK modeling can provide a precise initial estimate of expected outcomes without the need for actual dosing in rare patient populations. This approach paves the way for improved drug safety in RDs, where patients are often treated with multiple medications simultaneously. Moreover, this approach might also reduce the need for costly clinical interaction trials as demonstrated by the PBPK modeling approach presented by Lee et al. in their attempt to assess the DDI potential of vatiquinone for the treatment of Friedrich´s ataxia [60]. Coupling these PBPK models with disease progression models may offer a framework for more detailed and precise DTs in future development.

DTs are especially useful in the sense of drug repurposing in the context of RD research, as they simulate individual patients and therefore enable PK predictions of repurposed drugs without physical trials. However, although efficacy and safety assessments have been previously tested, and therefore drug repurposing is a valid approach to overcome research barriers in RD such as limited trial population, none of the included studies addressed this specifically. Nevertheless, as only 16 studies met the inclusion criteria, generalizability is limited. This review reflects the early stage of DT implementation in RD research despite its considerable promise and thus limits the possibility of evaluating effectiveness, safety and pathways for clinical adoption. It is also acknowledged that it is currently not possible to draw firm conclusions about the ability of DTs to accelerate RD research by, for example, overcoming typical challenges such as small trial populations, limited funding, and incomplete pathophysiological understanding to this time.

### 4.1 Barriers of DT implementation in clinical practice

Routine clinical implementation of DTs remains limited across healthcare and particularly scarce in RDs. Katsoulakis et al. review current deployments of DTs in healthcare such as the FDA’s Virtual Imaging Clinical Trials for Regulatory Evaluation (VICTRE) in breast imaging and Twin Health’s randomized trial (NCT05181449) for type-2 diabetes [88]. Most DT implementations are pilot projects or narrowly scoped, domain-specific initiatives rather than broadly accessible technologies for healthcare providers. DTs perform best in well-defined, data-rich, physiology-grounded settings (e.g., in-silico control arms, structured tumors or cardiovascular workflows, intensive remote monitoring) where inputs are consistent and outcomes are measurable [88]. As stated in chapter 1.2, measurable outcomes in RDs typically require costly methods and are a time-consuming practice as standardized assessments such as blood pressure measurement rarely exist for RDs.

The main barriers for broad DT implementation in clinical practice include data interoperability, ontologies, and standards. For instance, the field still lacks a consistent definition of “DT” and although standardized formats such as Fast Healthcare Interoperability Resources (FHIR), Digital Imaging and Communications in Medicine (DICOM), and Observational Medical Outcomes Partnership (OMOP) are available, heterogeneous data implementation continues to hinder the development of computable and shareable datasets required for DTs [88]. Further challenges include computational cost, because sustained simulation plus streaming analytics demands significant infrastructure, and interpretability as well as clinician acceptance. Moreover, rigorous validation against clinical evidence and reproducibility remain an open issue. Complementing this picture, Darwich et al. highlight concerns for MIPD. Clinicians are uncertain about responsibility when model-recommended doses diverge from the drug label, reinforcing that such tools must support, rather than replace, clinical judgment [89].

In RDs these challenges are amplified by limited and potentially biased data and additional ethical considerations (for example, how to construct a DT for a pregnant person with EB if available data are primarily from male patients or minors). Because RD research is already underfunded, broad adoption will require compelling clinical and economic evidence in addition to technical feasibility. DT implementation in clinical practice is therefore still at a very early stage in common disorders and even more in RDs.

### 4.2 Data accessibility for DT development in RDs

DTs are only as reliable as the data they are built upon. This poses a major challenge, as data sharing is often impeded by privacy regulations and a lack of incentives for collaboration. Therefore, this serves as a call to action for clinicians, pediatricians, RD networks, and researchers to make their findings, such as case studies, natural history data, PK measurements, biomarker data, and robust *in vivo* and *in vitro* results, accessible to the community. Access to high-quality data is essential for data scientists to build and refine their models which makes international and cross-institutional collaboration in data exchange between academia, industry and clinicians necessary. When the accessibility of model predictions for clinicians and caregivers is considered early in development, as demonstrated by Chelle et al., who created a user interface enabling patients to interact with the DT, patients can enter their own data and receive individualized dosing predictions. At the same time, modeling experts gain access to additional patient data, which can be used to further improve model predictions [63]. Here, attempts as seen by the CPI, a nonprofit organization dedicated to the collaboration of different stakeholders in the interest of research in RDs, are crucial to overcoming the problem of missing model input data and aligning on common standards [90]. This is especially relevant when attempting to extrapolate beyond available observations, such as predicting the probability of limb deformities occurring through extensive scarring in EB patients.

Since modeling requires detailed data, it highlights areas where further research and information are needed, ultimately advancing the understanding of the influence of disease (e.g. progression or cause) on clinical outcomes by revealing gaps in current knowledge. For example, causal disease modeling might address questions regarding disease severity in rare conditions such as EB: How does the genetic disruption in collagen VII, as seen in RDEB, correlate with disease severity? Which parameters influence variability among patients?

Emerging generative approaches, including Generative Adversarial Networks (GANs), Variational Autoencoders (VAEs), and Diffusion-based Models (DMs), can address the data gap and produce synthetic yet biologically plausible datasets, ranging from imaging and omics profiles to simulated patient trajectories [91], [92], [93]. In the RD context, these methods not only expand limited datasets and strengthen training pipelines but also support the development of DT frameworks. Beyond data augmentation, synthetic data generation can play a critical role in model validation and bias mitigation. By generating controlled synthetic cohorts that emulate specific demographic or clinical subgroups, these models enable systematic stress-testing of algorithms, detection of latent biases, and calibration of predictive performance across underrepresented populations [94]. Furthermore, integrating ontological or mechanistic priors into generative frameworks, such as ontology-enhanced GANs or knowledge-guided VAEs and DMs, can improve interpretability, biological consistency, and fairness, ultimately strengthening the translational reliability of AI-driven systems in RD research.

Recent work by Wu and Koelzer has expanded the DT concept beyond traditional mechanistic and pharmacometric frameworks toward generative DTs (GDTs) [95]. These GDTs integrate generative AI with omics and imaging-derived data, including spatial omics and multiplexed imaging, to create high-fidelity, spatially resolved virtual representations of biological systems. Unlike current RD DTs, which mainly rely on PBPK, PopPK, or QSP modeling, GDTs enable *in silico* causal interrogation (“what-if” simulations) by linking molecular and morphological data. This convergence of omics and diffusion- or GAN-based generative models opens a path toward data-driven, multi-scale DTs that can reproduce tissue and organ-level phenotypes with unprecedented realism. Incorporating such approaches into RD research could, in the long term, allow the generation of virtual cohorts that integrate omics-level perturbations with individualized physiological simulations, bridging current model-informed drug-development pipelines with next-generation AI-enabled DTs.

In conclusion, DTs can then assist in overcoming (i) limited patient availability and therefore difficulty in gold standard randomized clinical trial recruitment, (ii) difficulty in estimating individual disease progression and personal health outcomes, and (iii) substantial knowledge gaps in research of specific RDs accelerating safe, effective therapies for people living with RDs.

## CRediT authorship contribution statement

**Charlotte Maria Ursula Dette:** Writing – original draft, Visualization, Investigation, Conceptualization. **Veronika Alberg:** Writing – original draft, Visualization, Investigation, Conceptualization. **Simeon Rüdesheim:** Writing – review & editing, Project administration. **Dominik Selzer:** Writing – review & editing, Project administration. **Fatima Zahra Marok:** Writing – review & editing, Project administration. **Nicola Luigi Bragazzi:** Writing – review & editing. **Laura Maria Fuhr:** Writing – review & editing, Visualization. **Søren Brunak:** Writing – review & editing. **Ewan R. Pearson:** Writing – review & editing. **Tobias Zahn:** Writing – review & editing. **Dimitra Kiritsi:** Writing – review & editing. **Matthias Schwab:** Writing - review & editing. **Thorsten Lehr:** Writing – review & editing, Supervision, Project administration, Conceptualization

## Funding

MS and SR were in parts supported by the Robert Bosch Stiftung Stuttgart, Germany. MS was also funded by the Deutsche Forschungsgemeinschaft (DFG, 10.13039/501100001659German Research Foundation) under Germany’s Excellence Strategy – EXC 2180 - 390900677.

TL was supported by the SafePolyMed project which receives funding from the European Union’s Horizon Europe Research and Innovation Programme under Grant Agreement No. 101057639. Views and opinions expressed are, however, those of the author(s) only and do not necessarily reflect those of the European Union or the Health and Digital Executive Agency. Neither the European Union nor the granting authority can be held responsible for them.

For all authors, no specific funding was received for this work.

## Declaration of Competing Interest

The authors declare the following financial interests/personal relationships which may be considered as potential competing interests: Prof. Matthias Schwab, PhD reports financial support was provided by Robert-Bosch Foundation GmbH. Dr. Simeon Ruedesheim, PhD reports financial support was provided by Robert-Bosch Foundation GmbH. Prof. Matthias Schwab, PhD reports financial support was provided by German Research Foundation. Co-Author Dr. Tobias Zahn is shareholder and managing director of Crowd Pharma GmbH. If there are other authors, they declare that they have no known competing financial interests or personal relationships that could have appeared to influence the work reported in this paper.
