# Supplementary material for: Advancing rare disease therapeutics through digital twins: Opportunities in drug development and precision dosing
Source: Comput Struct Biotechnol J. 2025 Nov 23;28:592–608. doi: 10.1016/j.csbj.2025.11.047 (PMC12703978; doi:10.1016/j.csbj.2025.11.047)
Supplement: Supplementary file 1 — Supplementary material [file mmc1.docx]

Advancing Rare Disease Therapeutics Through Digital Twins: Opportunities in Drug Development and Precision Dosing

Supplementary Materials

Charlotte Maria Ursula Dette*^1^, Veronika Alberg*^1^, Simeon Rüdesheim^1,2^, Dominik Selzer^1^, Fatima Zahra Marok^1^, Nicola Luigi Bragazzi^1^, Laura Maria Fuhr^1^, Søren Brunak^3^, Ewan R. Pearson^4^, Tobias Zahn^5^, Dimitra Kiritsi^6,7^, Matthias Schwab^2,8,9^, Thorsten Lehr^1^

^1^ Clinical Pharmacy, Saarland University, 66123 Saarbrücken, Germany

^2^ Dr. Margarete Fischer-Bosch-Institute of Clinical Pharmacology, 70376 Stuttgart, Germany

^3^ Novo Nordisk Foundation Center for Protein Research, Faculty of Health and Medical Sciences, University of Copenhagen, Copenhagen, Denmark

^4^ Division of Diabetes, Endocrinology and Reproductive Biology, Ninewells Hospital and School of Medicine, University of Dundee, Dundee, UK

^5^ Crowd Pharma GmbH, 75179 Pforzheim, Germany

^6^ Department of Dermatology, Medical Center – University of Freiburg, Faculty of Medicine, University of Freiburg, 79106 Freiburg, Germany

^7^ First Department of Dermatology, Faculty of Medicine, Aristotle University of Thessaloniki, Thessaloniki, Greece

^8^ Departments of Clinical Pharmacology, Pharmacy and Biochemistry, University of Tübingen, 72076 Tübingen, Germany
^9^ Cluster of Excellence iFIT (EXC2180) “Image-Guided and Functionally Instructed Tumor Therapies”, University of Tübingen, 72076 Tübingen, Germany

* C.M.U. Dette and V. Alberg contributed equally to this work and are listed as co-first authors.

**Funding:**

MS and SR were in parts supported by the Robert Bosch Stiftung Stuttgart, Germany. MS was also funded by the Deutsche Forschungsgemeinschaft (DFG, German Research Foundation) under Germany’s Excellence Strategy – EXC 2180 – 390900677.

For all authors no specific funding was received for this work.

**Declaration of Interests:**

All authors declare no competing interest for this work.

**Corresponding Author:**

Prof. Dr. Thorsten Lehr, thorsten.lehr@uni-saarland.de

Clinical Pharmacy, Saarland University, Campus C4 3, 66123 Saarbrücken, Germany.

ORCID: 0000 0002 8372 1465

Phone: +49 681 302 70255

# S1 Digital Twins in Healthcare

## S1.1. Pubmed Search Terms

| **Table S1: Literature search term and additional filter criteria using the PubMed database (search performed on 24^th^ of July 2025).** | | | | |
| --- | --- | --- | --- | --- |
| Nr. | Search term |  |  | Additional criteria |
| 1 | ("digital twin*" OR "virtual patient*") | AND | ("healthcare" OR "medicine" OR "clinical care" OR "health services" OR "patient care" OR "medical care" OR "digital health" OR "public health" OR "precision medicine") | Language = English |

# S2 Methodology of Literature Research

## S2.1. Details of the Included Studies

**Table S2: Details of the studies included in the full-text review analyses**

| PMID | Title | First Author | Publication Year | DOI |
| --- | --- | --- | --- | --- |
| 40614125 | Quantitative Systems Pharmacology Modeling of Platelet Responses to Recombinant ADAMTS13 in Patients With Congenital Thrombotic Thrombocytopenic Purpura | McBride C | 2025 | 10.1002/psp4.70063 |
| 39612158 | Population Pharmacokinetic and Pharmacokinetic/Pharmacodynamic Analyses of Pegcetacoplan in Patients with Paroxysmal Nocturnal Hemoglobinuria | Crass RL | 2024 | 10.1007/s40268-024-00500-7 |
| 39372210 | A quantitative systems pharmacology (QSP) platform for preclinical to clinical translation of in-vivo CRISPR-Cas therapy | Desai DA | 2024 | 10.3389/fphar.2024.1454785 |
| 39308341 | Determination of Vatiquinone Drug-Drug Interactions, as CYP450 Perpetrator and Victim, Using Physiologically Based Pharmacokinetic (PBPK) Modeling and Simulation | Lee L | 2025 | 10.1002/jcph.6133 |
| 38698539 | Development of a Plasminogen Population PK model supporting prophylactic replacement therapy for Plasminogen deficient patients within the WAPPS-Hemo platform | Chelle P | 2024 | 10.1111/hae.15027 |
| 38372445 | A Generative and Causal Pharmacokinetic Model for Factor VIII in Hemophilia A: A Machine Learning Framework for Continuous Model Refinement | Janssen A | 2024 | 10.1002/cpt.3203 |
| 36653728 | Model-Informed Approach Supporting Approval of Nexviazyme (Avalglucosidase Alfa-ngpt) in Pediatric Patients with Late-Onset Pompe Disease | Li RJ | 2023 | 10.1208/s12248-023-00784-8 |
| 36056771 | Physiologically-Based Pharmacokinetic Model Development, Validation, and Application for Prediction of Eliglustat Drug-Drug Interactions | Sahasrabudhe SA | 2022 | 10.1002/cpt.2738 |
| 35118559 | Efficacy and safety exposure-response analyses of entrectinib in patients with advanced or metastatic solid tumors | Mercier F | 2022 | 10.1007/s00280-022-04402-w |
| 34146682 | Development and verification of an endogenous PBPK model to inform hydrocortisone replacement dosing in children and adults with cortisol deficiency | Bonner JJ | 2021 | 10.1016/j.ejps.2021.105913 |
| 32419339 | Leveraging Quantitative Systems Pharmacology Approach into Development of Human Recombinant Follistatin Fusion Protein for Duchenne Muscular Dystrophy | Nguyen HQ | 2020 | 10.1002/psp4.12518 |
| 30887238 | Simulating the Impact of Elevated Levels of Interleukin-6 on the Pharmacokinetics of Various CYP450 Substrates in Patients with Neuromyelitis Optica or Neuromyelitis Optica Spectrum Disorders in Different Ethnic Populations | Machavaram KK | 2019 | 10.1208/s12248-019-0309-y |
| 29410461 | In silico clinical trials for pediatric orphan diseases | Carlier A | 2018 | 10.1038/s41598-018-20737-y |
| 39707136 | Computer-generated Clinical Decision-making in the Treatment of Pulmonary Atresia with Intact Ventricular Septum | Yıldırım C | 2025 | 10.1007/s13239-024-00769-4 |
| 39632463 | Quantitative Systems Pharmacology-Based Digital Twins Approach Supplements Clinical Trial Data for Enzyme Replacement Therapies in Pompe Disease | Kaddi C | 2025 | 10.1002/cpt.3498 |
| 36908269 | Systems-based digital twins to help characterize clinical dose-response and propose predictive biomarkers in a Phase I study of bispecific antibody, mosunetuzumab, in NHL | Susilo ME | 2023 | 10.1111/cts.13501 |
| In silico CT: in silico clinical trials, PBPK: physiologically based pharmacokinetic modeling, PD: pharmacodynamic modeling, PopPK: population pharmacokinetic modeling, Prec. Data: preclinical data, QSP: quantitative systems pharmacology, Reg. Approv.: regulatory approval, Scal. Children: scaling to children | | | | |

## S2.2. Overview of the Included Studies

Table S3: Overview of the included studies with information on model type, validation method, data source, key outcome and criteria met in context of DT.

| Reference | Model Type | Validation Method | Data Source | Key Outcome |
| --- | --- | --- | --- | --- |
| McBride et al. | QSP, PD, *in silico* CT, Reg. Approv., Prec. Data | data from patients with cTTP receiving infusions of fresh frozen plasma as standard of care: measured platelet production rate and baseline VWF level were compared with the model prediction, 15 patients from Phase 3 cTTP study used as validation dataset | *in vitro*, *adamts13*-knockout mouse, literature-based, clinical data | clinical benefit of rADAMTS13: reduce occurrences of thrombocytopenia in cTTP patients 🡪 confirmative evidence, significant potential of QSP models to supplement clinical trial data in rare disease drug development |
| Crass et al. | PopPK, PD | NA | clinical data (pooled) from 11 completed studies in different patient groups (healthy, PNH, renal impaired) | robust biomarker responses across different body weights and no influence of eculizumab treatment history 🡪 no pegcetacoplan initial dose adjustments needed |
| Desai et al. | QSP, PD, Prec. Data | NA | published literature | successful preclinical-to-clinical translation of in vivo CRISPR-Cas therapies with dose-response relationship |
| Lee et al. | PBPK | one DDI study (rifampicin) and one Phase 2 patient study | *in vitro*, *in silico*, physiochemical and clinical data | addressing DDI gaps without the need for conducting additional studies, interaction risk coadministration with CYP3A4 perpetrators |
| Chelle et al. | PopPK | NA | Retrospective data collected from participants included in two clinical trials | integration of the PopPK model into the WAPPS-Hemo database |
| Janssen et al. | PopPK | external data set of patients who received octocog alfa and turoctocog alfa | literature | prediction of FVIII levels in patients with hemophilia A, approach can be used to generate missing data, sensitive data can be made publicly available |
| Li et al. | PopPK, *in silico* CT, Reg. Approv., Scal. Children | NA | clinical studies (3 in LOPD patients, 1 in IOPD patients) | approval support of a model-informed dosing regimen of avalglucosidase alfan-gpt in pediatric patients with LOPD, without conducting an additional pediatric trial |
| Sahasrabudhe et al. | PBPK | external dataset | published literature | simulating clinically untested DDI scenarios, supporting dose finding |
| Machavaram et al. | PBPK | NA | published literature | elevated IL-6 levels in NMO/NMOSD patients may significantly reduce the metabolism of CYP450 substrates (except CYP1A2)🡪 increasing substrate exposure |

**Table S3: Overview of the included studies with information on model type, validation method, data source, key outcome and criteria met in context of DT. (*continued*)**

| Reference | Model Type | Validation Method | Data Source | Key Outcome |
| --- | --- | --- | --- | --- |
| Mercier et al. | PopPK | NA | clinical studies (3 studies in total) | doses equal to or exceeding 800 mg QD showed higher frequency of Grade ≥ 3 TEAE or an SAE, exposure-efficacy analysis showed no gradient of response across the range of entrectinib concentration 🡪 an entrectinib dose of 600 mg QD can provide an acceptable balance between efficacy and safety in adults |
| Bonner et al. | PBPK, Scal. Children | clinical studies | published literature, clinical studies in patients with cortisol deficiency and different formulations | Development of hydrocortisone PBPK model in adults and children with ontogeny and different formulations, supports dose optimization for cortisol replacement therapy |
| Nguyen et al. | QSP, PD, Prec. Data | predicting muscle volume changes for ACE-031 and domagrozumab (not used for development) | Preclinical and clinical data, experimental *in vitro* and *in vivo* data, published literature | model informs dual-target efficacy, binding affinity needs, and predicted human dose-response. Weekly 3 to 5 mg/kg follistatin-Fc dosing could achieve ~7 to 10 % muscle volume increase in Duchenne patients |
| Carlier et al. | PD, *in silico* CT | NA | published literature | simulation predicted lower complication index with BMP vs. control and identified four patient-response subgroups, highlighting potential biomarkers of treatment response |
| Yıldırım et al. | *in silico* CT | published literature, patient data | clinical studies, published literature | for patients with RV size > 22 mL/m², biventricular repair was likely successful; about 30 % of virtual patients failed biventricular repair and needed 1.5-ventricle, and ~14 % of those still failed post-1.5-ventricle repair |
| Kaddi et al. | QSP, PD, *in silico* CT | clinical studies, biomarker data, tissue data | published natural history and real-world data, clinical studies, preclinical data | residual enzymatic activity of the endogenous GAA reaction as key parameter calibrated to generate digital twins of clinical patients 🡪 greater urinary Hex4 reduction with avalglucosidase alfa due to improved glycogen clearance; approach supports interpretation of clinical outcomes |
| Susilo et al. | QSP, PD, *in silico* CT | clinical studies | clinical studies, preclinical data, *in vitro* data, *in vivo* data, published data | higher exposure increased proportion of digital twins with ≥50% tumor reduction, indolent NHL patients displayed greater sensitivity due to lower tumor proliferation and higher T-cell infiltration 🡪 using QSP for prediction of response and biomarker identification |

ADAMTS13: a disintegrin and metalloproteinase with thrombospondin type 1 motif, member 13, BMP: bone morphogenetic protein, cTTP: congenital thrombotic thrombocytopenic purpura, CRISPR-Cas: clustered regularly interspaced short palindromic repeats-CRISPR-associated, CYP: cytochrome P450, DDI: drug-drug interaction, DT: digital twin, FVIII: factor VIII, follistatin-Fc: follistatin fusion protein (Fc-tag), GAA: acid alpha-glucosidase, HEX4: hydroxyethyl-lysine 4, IL: interleukin, *in silico* CT: *in silico* clinical trials, IOPD: infantile-onset Pompe disease, LOPD: late-onset Pompe disease, NA: not applicable, NHL: non-Hodgkin lymphoma, NMO: neuromyelitis optica, NMOSD: neuromyelitis optica spectrum disorder, PBPK: physiologically based pharmacokinetic modeling, PD: pharmacodynamic modeling, PopPK: population pharmacokinetic modeling, PNH: paroxysmal nocturnal hemoglobinuria, QD: once daily, QSP: quantitative systems pharmacology, rADAMTS13: recombinant ADAMTS13, RV: right ventricle, SAE: serious adverse event, TEAE: treatment-emergent adverse event, VWF: von Willebrand factor, WAPPS-Hemo: web-accessible population pharmacokinetic service-hemophilia.

## S2.3. Existing Reviews Related to Digital Twins and Rare Diseases

The literature search was performed on July 8^th^, 2025 using the PubMed database. The search initially retrieved 49 articles. 16 of these articles were reviews, that were excluded from further full-text analyses. The excluded reviews are presented in Table S4.

Table S4: Overview of the reviews in the field of DTs and RDs.

| PMID | Title | First Author | Publication Year | DOI |
| --- | --- | --- | --- | --- |
| 37360227 | A systematic review of cardiac in-silico clinical trials | Rodero C | 2023 | 10.1088/2516-1091/acdc71 |
| 34966890 | Computational Approaches for Supporting Combination Therapy in the Post-Aducanumab Era in Alzheimer's Disease | Geerts H | 2021 | 10.3233/ADR-210039 |
| 34103684 | How artificial intelligence might disrupt diagnostics in hematology in the near future | Walter W | 2021 | 10.1038/s41388-021-01861-y |
| 27943015 | Personalized Drug Dosage - Closing the Loop | Tucker GT | 2016 | 10.1007/s11095-016-2076-0 |
| 40560515 | Model-Informed Precision Dosing of Eculizumab in Patients with Paroxysmal Nocturnal Hemoglobinuria | Ter Avest M | 2025 | 10.1007/s40262-025-01536-x |
| 37984065 | Creating a Roadmap to Quantitative Systems Pharmacology-Informed Rare Disease Drug Development: A Workshop Report | Bai JP | 2023 | 10.1002/cpt.3096 |
| 37429704 | The new big is small: Leveraging knowledge from small trials for rare disease drug development: Blarcamesine for Rett syndrome | Ette El | 2023 | 10.1111/bcp.15843 |
| 34704362 | Model-informed assessment of ethnic sensitivity and dosage justification for Asian populations in the global clinical development and use of cladribine tablets | Munafo A | 2021 | 10.1111/cts.13166 |
| 39340225 | Quantitative Systems Pharmacology Models: Potential Tools for Advancing Drug Development for Rare Diseases | Neves-Zaph S | 2024 | 10.1002/cpt.3451 |
| 39145605 | Pediatric Rare Diseases Development in the Pharmaceutical Industry: An International Consortium for Innovation and Quality in Pharmaceutical Development, Clinical Pharmacology Leadership Group-Pediatrics Working Group, Rare Diseases Subteam Whitepaper Examining the Current Landscape and Recommendations for the Future | Krishna R | 2024 | 10.1002/cpt.3422 |
| 37422281 | Quantitative Systems Pharmacology for Rare Disease Drug Development | Bai JP | 2023 | 10.1016/j.xphs.2023.06.019 |
| 37317497 | Supporting Prospective Pregnancy Trials via Modeling and Simulation: Lessons From the Past and Recommendations for the Future | Cheung SYA | 2023 | 10.1002/jcph.2284 |
| 37296230 | Challenges, approaches and enablers: effectively triangulating towards dose selection in pediatric rare diseases | Durairaj C | 2023 | 10.1007/s10928-023-09868-6 |
| 36461744 | Model-Informed Approach Supporting Drug Development and Regulatory Evaluation for Rare Diseases | Li RJ | 2022 | 10.1002/jcph.2143 |
| 34860361 | Clinical Pharmacology in Drug Development for Rare Diseases in Neurology: Contributions and Opportunities | Abuasal B | 2022 | 10.1002/cpt.2501 |
| 30465453 | Dose adjustment in orphan disease populations: the quest to fulfill the requirements of physiologically based pharmacokinetics | Howard M | 2018 | 10.1080/17425255.2018.1546288 |
